# Imaging of fibrogenesis in the liver by [^18^F]TZ-Z09591, an Affibody molecule targeting platelet derived growth factor receptor β

**DOI:** 10.1186/s41181-023-00210-6

**Published:** 2023-09-21

**Authors:** Olivia Wegrzyniak, Bo Zhang, Johanna Rokka, Maria Rosestedt, Bogdan Mitran, Pierre Cheung, Emmi Puuvuori, Sofie Ingvast, Jonas Persson, Helena Nordström, John Löfblom, Fredrik Pontén, Fredrik Y. Frejd, Olle Korsgren, Jonas Eriksson, Olof Eriksson

**Affiliations:** 1 Science for Life Laboratory, Department of Medicinal Chemistry, Uppsala University, Dag Hammarskjölds Väg 14C, 3tr, 751 83 Uppsala, Sweden; 2 Department of Immunology, Genetics and Pathology, Uppsala University, Uppsala, Sweden; 3 Antaros Medical AB, Uppsala, Sweden; 4 Department of Protein Science, Division of Protein Engineering, KTH Royal Institute of Technology, Stockholm, Sweden; 5 Affibody AB, Solna, Sweden; 6 Science for Life Laboratory, Drug Discovery and Development Platform, Department of Chemistry-BMC, Uppsala University, Uppsala, Sweden; 7 Uppsala University Hospital PET Center, Entrance 85, Dag Hammarskjölds Väg 21, 752 37 Uppsala, Sweden

**Keywords:** PET imaging, Platelet derived growth factor receptor, Hepatic stellate cells, Fibrogenesis, Liver fibrosis

## Abstract

**Background:**

Platelet-derived growth factor receptor beta (PDGFRβ) is a receptor overexpressed on activated hepatic stellate cells (aHSCs). Positron emission tomography (PET) imaging of PDGFRβ could potentially allow the quantification of fibrogenesis in fibrotic livers. This study aims to evaluate a fluorine-18 radiolabeled Affibody molecule ([^18^F]TZ-Z09591) as a PET tracer for imaging liver fibrogenesis.

**Results:**

In vitro specificity studies demonstrated that the trans-Cyclooctenes (TCO) conjugated Z09591 Affibody molecule had a picomolar affinity for human PDGFRβ. Biodistribution performed on healthy rats showed rapid clearance of [^18^F]TZ-Z09591 through the kidneys and low liver background uptake. Autoradiography (ARG) studies on fibrotic livers from mice or humans correlated with histopathology results. Ex vivo biodistribution and ARG revealed that [^18^F]TZ-Z09591 binding in the liver was increased in fibrotic livers (p = 0.02) and corresponded to binding in fibrotic scars.

**Conclusions:**

Our study highlights [18F]TZ-Z09591 as a specific tracer for fibrogenic cells in the fibrotic liver, thus offering the potential to assess fibrogenesis clearly.

**Graphical abstract:**

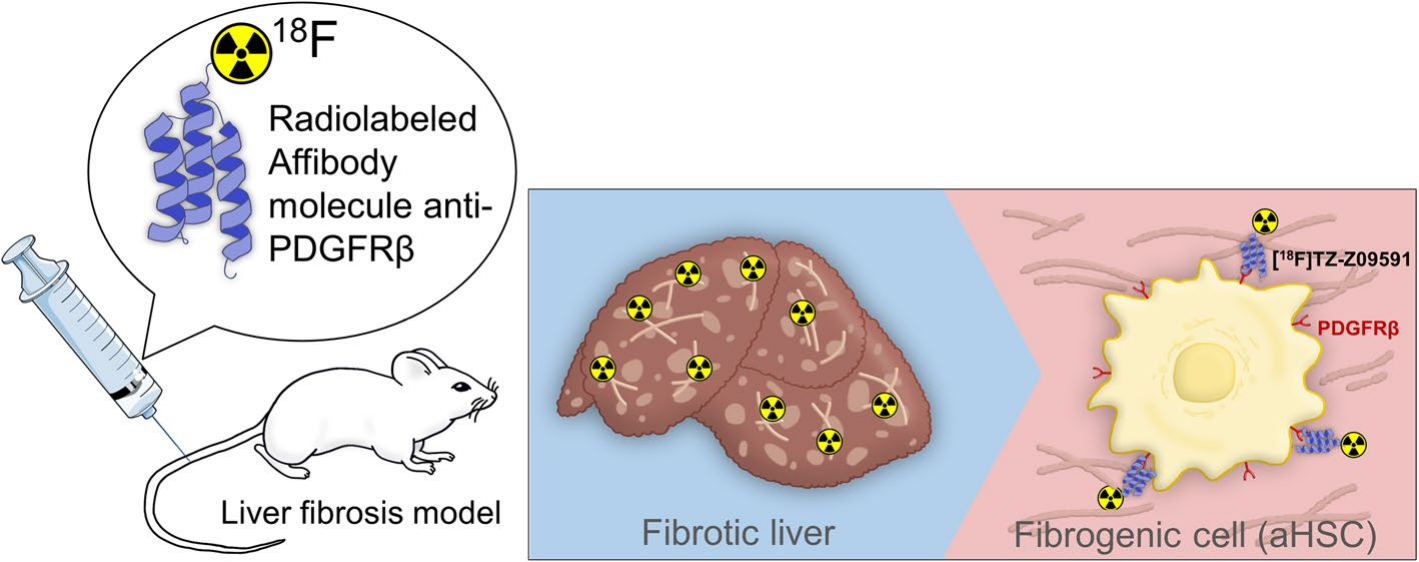

**Supplementary Information:**

The online version contains supplementary material available at 10.1186/s41181-023-00210-6.

## Background

Fibrogenesis is a dynamic process characterized by the ongoing accumulation of extracellular matrix (ECM), which can lead to the dysfunction or failure of the affected organ. Fibrotic diseases can occur in any organ, including the liver. Liver fibrosis can have several etiologies, but today those of metabolic origin are considered an important societal challenge (Younossi et al. 2016; Haldar et al. 2019) because of the increasing prevalence of obesity and diabetes (Worldwide trends in body-mass index 2017; Zhou et al. 2016).

Today, liver fibrosis is rarely diagnosed before an advanced stage due to the lack of significant clinical signs associated with a moderate stage. Unfortunately, no anti-fibrotic therapies are currently approved in the case of severe liver fibrosis. Recently, efforts have been made to develop non-invasive tests, such as serum assay (e.g. FibroTest) (EASL-ALEH Clinical Practice Guidelines 2015), imaging tests with, for instance, elastography devices (Parker et al. 1998; Barr et al. 2016) (e.g. FibroScan) or multifactorial scoring systems (e.g. AST/platelet ratio index (APRI) and the Fibrosis Index Based on 4 Factors (FIB-4)) (Wai et al. 2003; Sterling et al. 2006; Lee et al. 2021). Unfortunately, these methods are still limited by their low sensitivity and specificity, especially at an early stage of the disease (EASL-ALEH Clinical Practice Guidelines 2015; Patel and Sebastiani 2020). The gold standard for assessing fibrosis is liver biopsy and its unavoidable limitations (invasiveness, complications, sampling variability, etc*.*). Therefore, the development of a non-invasive technique to quantify fibrogenesis would potentially be a major step forward in providing a more reactive and sensitive assessment of the activity of antifibrotic therapies.

Hepatic stellate cells (HSCs) when activated (aHSCs) play a major role in liver fibrogenesis as they are known to be the main source of ECM in liver fibrosis (Friedman et al. 1985). There is an interest in imaging aHSCs as such an approach has the potential for diagnosis at an early stage, before pathological changes, prognosis, and monitoring reactively the response to treatment (Li et al. 2015).

Platelet-derived growth factor receptor beta (PDGFRβ) is a biomarker of aHSCs but is absent on resting HSCs (Pinzani et al. 1996; Wong et al. 1994), it is thus a biomarker of fibrogenesis in the liver. The Affibody molecule Z09591 targeting PDGFRβ with a subnanomolar affinity was previously developed(Lindborg et al. 2011). Affibody molecules are small molecules of 58-amino-acid (6.5 kDa) three-helical bundle polypeptide. They are robust, easy to engineer, and small relative to *e.g.* antibodies, which is favorable for diagnostic imaging due to their rapid biodistribution and tissue penetration(Frejd and Kim 2017). The Affibody molecule Z09591 was previously labelled with a dodecane tetraacetic acid (DOTA) chelator and evaluated for the molecular imaging of PDGFRβ-expressing xenografts with [^111^In]DOTA-Z09591 and [^68^ Ga]DOTA-Z09591(Tolmachev et al. 2014; Strand et al. 2014). However, labeling with fluorine-18 would be of interest since it has ideal nuclear properties for positron emission tomography (PET) imaging, allowing for *e.g.* high-resolution images as well as facile logistics due to its two-hour radioactive half-life.

Hence, given the implication of PDGFRβ in fibrogenesis and given the characteristics of the Affibody molecule Z09591, we speculated that the radiolabeled Affibody molecule targeting PDGFRβ could be suitable for detecting liver fibrogenesis. To further this hypothesis, the Affibody molecule Z09591 was conjugated to a trans-cyclooctene (TCO) group and labeled to a Flourine-18 labeled tetrazine (TZ) group, the resulting radiotracer [^18^F]TZ-Z09591 was evaluated in vitro on liver biopsies from patients with liver fibrosis and in vivo using the carbon tetrachloride (CCl_4_) mouse model of liver fibrosis.

## Methods

### Affibody molecule

The sequence of Affibody molecule Z09591 was described previously(Lindborg et al. 2011; Tolmachev et al. 2014; Strand et al. 2014). Z09591 was produced in a minimized format only containing the three-helix Affibody scaffold (58 amino acids, including the PDGFRβ binding motif) by chemical solid phase peptide synthesis (Almac). A unique cysteine residue was added to the C-terminal, which was conjugated with a TCO group via a tris-polyethylene glycol (PEG_3_) linker. The resulting precursor (TCO-conjugated Z09591) was purified by Reverse phase high performance liquid chromatography (HPLC), and freeze-dried in aliquots of 100µg each. Identity was verified by Mass Spectrometry.

### Radiochemistry

**Fig. 1:**
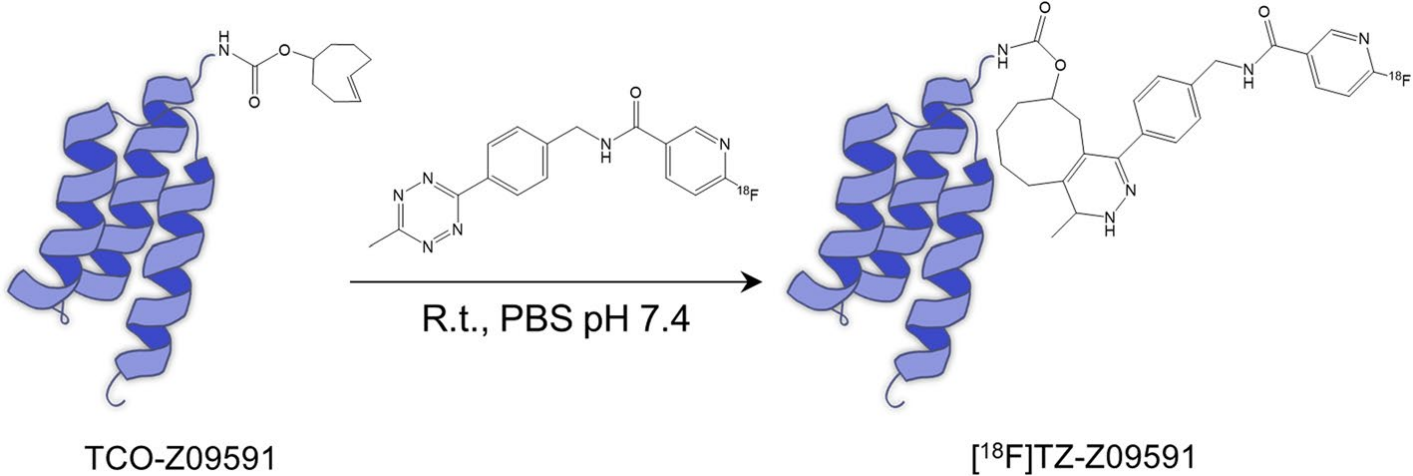
[^18^F]TZ-Z09591 structure. Schematic presentation of the molecular structure of the conjugated [^18^F]TZ-Z09591

[^18^F]MeTZ was radiolabeled by a prosthetic flourine-18 labeled TZ group via a click chemistry procedure (Fig. 1) previously described for radiolabeling of proteins (Syvänen et al. 2020).

#### Synthesis of [^18^F]MeTz

[^18^F]Fluoride in an aqueous solution was trapped and concentrated on a Chromabond PS-HCO3 Shorty/45 mg cartridge (Macherey–Nagel). The cartridge-bound [^18^F]fluoride was reacted with a solution of precursor N,N,N-trimethyl-5-((2,3,5,6-tetrafluorophenoxy)carbonyl)pyridin-2-aminium trifluoromethanesulfonate (10 mg) in acetonitrile (0.8 mL). This was followed by elution with neat acetonitrile (0.7 mL). Both solutions at a flow rate of 0.4 mL/min. The resulting product, [^18^F]F-Py-TFP, formed instantly at room temperature and was collected in a Teflon tubing. The product-containing solution from the tubing was pushed with air through an Oasis MCX Plus Short cartridge (Waters) pre-conditioned with acetonitrile (3 mL), ensuring removal of any unreacted precursor 1.The purified [^18^F]F-Py-TFP was introduced to a septum-equipped vial (5 mL) holding (4-(6-methyl-1,2,4,5-tetrazin-3-yl)phenyl)methanamine hydrochloride 2 (1.0 mg) predisolved in DMSO (0.1 mL) to enhance solubility. Subsequently, a solution of triethylamine (5 µL) in acetonitrile (100 µL) was added, and the mixture was heated for 15 min at 55°C.

Post heating, the reaction mixture was diluted with aqueous trifluoroacetic acid (3 mL, 0.1%). The labelled tetrazine, [^18^F]MeTz, was isolated using semi-preparative HPLC on an ACE C18 5 µm (150 × 10 mm) column. The mobile phase consisted of water, ethanol, and TFA (62:38:0.01%) with a flow rate of 5 mL/min. Detection was achieved with a UV detector (254 nm) and a radiodetector. The retention time of [^18^F]MeTz was 8.5 min.

The collected HPLC fraction containing [^18^F]MeTz was reformulated in ethanol (0.5 mL) using a conventional procedure. This process included dilution with water (20 mL), adsorption on a Sep-Pak tC18 Plus Light Cartridge cartridge, a rinse with water (20 mL), and air prior elution with the ethanol (0.5 mL) into a septum equipped vial (1 ml). The ethanol was the then removed by evaporation under a nitrogen gas stream and heating at 65°C for 7 min, resulting in [^18^F]MeTz with minimal residual water droplets in the vial.

The radiochemical purity of [^18^F]MeTz was assessed by analytical HPLC using a Kinetex C18 column (2.6 µm 100 Å, 100 × 3.0 mm, Phenomenex). The elutent was had a 75:25 water to acetonitrile ratio, with a flow rate of 0.7 mL/min giving a 6.6-min retention time for [^18^F]MeTz. The identity of the radiolabelled tetrazine was verified by co-injecting and comparing retention times with an authentic standard. The molar activity of [^18^F]MeTz was assessed by quantifying the tracer concentration via the same HPLC method and measuring the activity using a dose calibrator.

#### Synthesis of [^18^F]TZ-Z09591

A solution of TCO-functionalized Z09591 Affibody molecule (100 µg, 14 nmol) in phosphate-buffered saline (PBS) (300 µL) was introduced to a vial containing [^18^F]MeTz (2.0 ± 0.6 GBq). The mixture was allowed to react at room temperature for 10–15 min to produce [^18^F]TZ-Z09591. The labeled Affibody molecule was purified from unreacted [^18^F]MeTz using size exclusion chromatography on a NAP-5 column (Cytiva). After preconditioning the column with PBS and 10% ethanol (two column volumes), the product mixture was loaded. Elution was performed with PBS (1 mL). The purified [^18^F]TZ-Z09591 was obtained in the eluate (approximately 1 mL) while [^18^F]MeTz remained on the column.

The radiochemical purity of [^18^F]TZ-Z09591 was assessed using analytical HPLC on a Vydac 214MS C4 column (5 µm, 300 Å, 50 × 4.6 mm, Phenomenex). The elution system consisted of water with 0.1% TFA and acetonitrile, using a gradient that increased from 5 to 80% acetonitrile over 10 min, at a flow rate of 4 mL/min. [^18^F]TZ-Z09591 exhibited a retention time of 3.5 min. The identity of the [^18^F]TZ-Z09591 Affibody molecule was confirmed by co-injecting and matching elution profiles with the TCO-conjugated DGCR2 Affibody molecule.

### Cell culturing

PDGFRβ positive U-87 human glioblastoma cells (ATCC) were used as a suitable model for binding studies using [^18^F]TZ-Z09591. PDGFRβ negative K-562 cells (ATCC) were used as a negative control (Table S1). Cells were cultured in Eagle’s minimum essential medium (ATCC) and in Roswell Park Memorial Institute (RPMI)-1640 (Biowest) respectively, complemented with 10% fetal bovine serum (Merck), and 1% Penicillin–Streptomycin (Biochrom). After culturing and expansion, the cells were washed in PBS and then frozen for sectioning for use in in vitro autoradiography (ARG) binding assays.

### Autoradiography

In order to study the specificity of [^18^F]TZ-Z09591 in vitro, ARG binding studies were performed on biopsies, and cell pellets sectioned with a cryostat microtome (Micron HM560), and mounted on glass slides. See supplemental materials for details on the protocol. The samples, optionally pre-treated with blocking compound, were incubated in a solution containing 5 nM of [^18^F]TZ-Z09591 (0.1 MBq/mL) for one-hour incubation at room temperature, followed by washes in cold PBS / 1% bovine serum albumin (BSA) solution and cold Milli-Q water. A reference consisting of a 10µL drop of the radiotracer-containing solution of known radioactivity was placed on an absorbent paper glued to a glass slide. The sections and reference were then placed in contact with a phosphor-imaging plate overnight. Once the exposure was complete, the plate was scanned by a phosphor-imager system (Amersham Typhoon IP, GE Healthcare). The ImageJ software (ImageJ 1.45S) was used to visualize and analyze the sections. The data obtained from in vitro ARG are expressed as fmol/mm3.

Ex vivo autoradiography follow the same protocol, except that the organs collected from animals injected with [^18^F]TZ-Z09591 were snap-frozen, and the sections obtain from these were directly placed in contact with a phosphor-imaging plate.

### Counterstaining

Formalin-fixed paraffin-embedded tissues were sectioned into 4 μm sections and stained with hematoxylin–eosin (H&E) and Sirius Red (SIR) according to standard methods at the local hospital pathology department (Uppsala University Hospital). Immunohistological stainings (IHC) were performed for: PDGFRβ expression using a recombinant anti-PDGFR antibody ((ab32570, Abcam) at 1:300; neutrophil elastase (NES) using an anti-NES antibody (ab68672, Abcam); cluster of differentiation 68 (CD68) using an anti-CD68 polyclonal antibody (ab125212, abcam) at1:100 (Table S2). All primary antibodies were incubated for 60 min, and horseradish peroxidase*** (***HRP)-conjugated secondary antibodies were used against the primary antibodies.

Sections were digitally imaged with a PathScan Enabler IV (Meyer Instruments) at 5.0 × magnification, and the images were visualized using QuPath-0.2.3.

### Human liver biopsies

Sections of frozen liver biopsies from patients with different stages of fibrosis (*n* = *9*) was obtained from the Uppsala Biobank (#827). The use of human biopsies for ARG binding studies was approved by the Swedish Ethical Review Authority (2019-02790). The frozen sections were used for ARG binding studies, while adjacent sections were stained for SIR at the Uppsala University Hospital pathology department. The SIR stained biopsies were submitted to a pathologist for blinded assessment of their fibrotic stage (F1, *n* = *3*; F2, *n* = *3*; F3, *n* = *3*).

### Animal welfare

Mice were housed at 5 per cage, and rats were 2 per cage, lined with bedding (GLP Aspen Bedding, Tapvei), in individually ventilated caging. All animals were kept under a constant temperature of 22 °C and humidity (50%), in a 12 h light/12 h dark rhythm and had access to rodent chow and water ad libitum*.*

Animal care was provided according to the local institutional (“Uppsala university guidelines on animal experimentation”, UFV 2007/724), national, and European Union animal care rules and regulations and conformed to the ARRIVE (Animal Research: Reporting of In Vivo Experiments) guidelines. All study protocols involving animal experimentation were approved by Animal Ethics Committee of the Swedish Animal Welfare Agency.

### Pharmacokinetics in healthy rats

Sixteen male (312.7 ± 18.3 g) Sprague Dawley rats were used for *ex vivo* organ distribution of the [^18^F]TZ-Z09591. They were administered into the tail vein with 3.15 ± 1.3 MBq and euthanized at different time points (*n* = *2* per time point): 5, 10, 30, 40, 60, and 120 min post-injection. The following organs were then collected, weighed and their radioactivity content measured in a gamma-counter: blood, heart, lungs, liver, pancreas, spleen, adrenal glands, kidneys, small intestines with or without content, large intestines without content, feces, urine, bladder, testis, muscle, bone, bone marrow, brain, and injection site (tail). Radioactivity in the remaining carcass was also measured. The radioactivity measured was corrected for decay and converted into standardized uptake values (SUVs) according to Eq. (1), the radioactivity is expressed in MBq and the weight in grams:

1 $${\text{SUV}} = \frac{{{\text{Radioactivity}}_{{{\text{organ}}}} *{\text{weight}}_{{{\text{rat}}}} }}{{{\text{Rdioactivity}}_{{{\text{injected}}}} *{\text{weight}}_{{{\text{organ}}}} }}$$

The resulting SUVs were used to determine organ and whole‐body effective doses (mSv/MBq) in humans as described by Velikyan et al. (2021).

Three rats had a misinjection, and the radiotracer remained in the tail. Thus, they were excluded from the study, leading to only one rat for the 5-, 10-, and 40 min time points. Moreover, the measurement of radioactivity content of the large intestines without content and the femur of one rat at 20 min could not be acquired because of instrumental errors.

Separately, an additional four male (265.9 ± 16.7 g) Sprague Dawley rats were examined by dynamic whole-body PET/magnetic resonance imaging (MRI) scanning. Each animal was anesthetized (0,6L/min of 3% sevoflurane in 50/50% oxygen/medical air,) and placed in prone position on the bed of a preclinical PET/MRI scanner (nanoPET, 3T field strength, 10 cm field of view, Mediso). A heated bed supported body temperature, maintaining it at 37°C. Positioning was assisted by a scout MRI scan (GRE MultiFOV scout). [^18^F]TZ-Z09591 (10.4 ± 1.3 MBq) was administered as a bolus through an intravenous catheter in the tail. At the same time, scanner acquisition was started and continued for 150 min. The PET protocol was designed to provide repeated whole-body passes of increasing duration (3 beds per pass; 2 × 5 min, 2 × 10min, 4 × 30 min). After the scan, the animal was euthanized and whole-body MRI images were acquired for anatomical co-registration (SE MultiFOV).

Whole-body PET/MRI images were visualized and analyzed using the PMOD software (PMOD Technologies). Briefly, tissues of interest (brain, blood, lung, liver, spleen, kidneys, urine bladder, bone, bone marrow, muscle, small intestine, lower and upper large intestine (lumen/ content was included for each intestinal segment), stomach content) were segmented on summarized PET images and MRI projections, and the PET uptake value for each time point read out as SUVmean. The entire body was also segmented.

### Evaluation in a fibrotic liver model

We studied liver fibrosis in a CCl_4_ mouse model. In a pilot experiment, BALB/c female mice (*n* = *8*) were treated with CCl_4_ (Sigma) at a dose of 0.5 mg/g of body weight in a 1:4 CCl_4_: corn oil mixture. The solution was administered to the lower side of the abdomen three times a week for three weeks. After three weeks of treatment, their liver was collected, as well as the liver of five healthy control mice, and divided into two biopsies: one frozen and one in paraformaldehyde (PFA) followed by embedding in paraffin. Sections from the paraffin-embedded biopsy were stained with SIR for fibrosis scoring, while the frozen biopsy was sectioned for in vitro ARG using [^18^F]TZ-Z09591 (see methodology above) as well as SIR staining of the same frozen section. However, the pilot study also produced generally weak and variable development of fibrosis and fibrogenesis, and we decided to increase the treatment duration for the full study.

Thus, in the full study, the mice (BALB/c female mice, *n* = *15*) were treated with CCl_4_ for six weeks instead of three. Three mice were euthanized before the end of the treatment as they reached the humane endpoint. In this study, healthy control mice (*n* = *6*) were also included. These mice were used to realize ex vivo biodistribution study, ex vivo and in vitro ARGs.

To ensure animal well-being, the mice were monitored closely for 30 min after each CCl_4_ injection and once per day.

After the induction of liver fibrosis, [^18^F]TZ-Z09591 was injected intravenously at a concentration corresponding to a peptide mass of 0,6 µg (1,25 ± 0,32 MBq) or 3 µg (7,70 ± 1,06 MBq). As part of the blocking study, some mice received a pre-injection of 40µg Cys-Z09591, 10 min before receiving the radiotracer injection. The detail of the different groups is described in the supplementary data (Additional file 1: Fig. S1).

During the 1-h [^18^F]TZ-Z09591 uptake time, mice were kept conscious in room air. One hour post-injection, the mice were euthanized, and blood was collected from heart puncture into a Lithium Heparin tube (Vacutest Kima) for biomarker analysis as described below. Then, the liver, spleen, and leg muscle were excised, to realize ex vivo biodistribution study, ex vivo, or in vitro ARGs, and histology studies.

### p-ALT and p-AST assay

Mouse plasma was collected via heart puncture at the time of euthanasia. Plasma alanine aminotransferase (ALT) and Aspartate transaminase (AST) levels were analyzed using enzyme-linked immunosorbent assays (ELISAs) according to standard methods at Uppsala University Hospital. The results are expressed as ukat/L.

### Statistical analysis

Results are presented as mean ± standard deviation (SD). Normality was checked using a Shapiro–Wilk test and equality of variance with a F-test. Data following a normal distribution and equal variances were analyzed using unpaired two-tailed Student's t-tests. If variances were not equal, an unpaired t-test with Welch’s correction was performed. If the data did not follow a normal distribution, the significance of the difference between the groups was analyzed using the Mann–Whitney test. Pearson’s tests were performed to assess the significance of linear correlations. For all comparisons, a significance level of p < 0.05 was applied. Statistical analyses were performed using GraphPad Prism 9.3.1 software (GraphPad Software).

## Results

### Radiochemistry

Starting from 8 to 15 GBq of [^18^F]fluoride the isolated radiochemical yield of [^18^F]MeTz was 18 ± 4% (*n* = *21*) and the radioactivity yield of [^18^F]TZ-Z09591 was 390 ± 80 MBq (*n* = *19*). The radiochemical purity was > 99%. [^18^F]MeTz was likely applied in molar excess given the extremely low molar amount of Affibody molecule (17 nmol) used in the reaction. This assumption was supported by the observation that 0.5–2.5 GBq of [^18^F]MeTz gave a similar amount of labeled Affibody molecule, indicating that all available TCO-groups were consumed. The molar activity at the end of synthesis was usually in the range of 20 MBq/nmol.

### In vitro stability

In vitro stability of [^18^F]TZ-Z09591 was evaluated in rat plasma, human plasma, and in PBS, pH 7.4. In plasma, no increase in decomposition was observed compared to that in PBS. A purity of approximately 90% was observed after 3h, and no difference between rat and human plasma and PBS was seen (Fig. S2).

### In vitro autoradiography binding studies

**Fig. 2:**
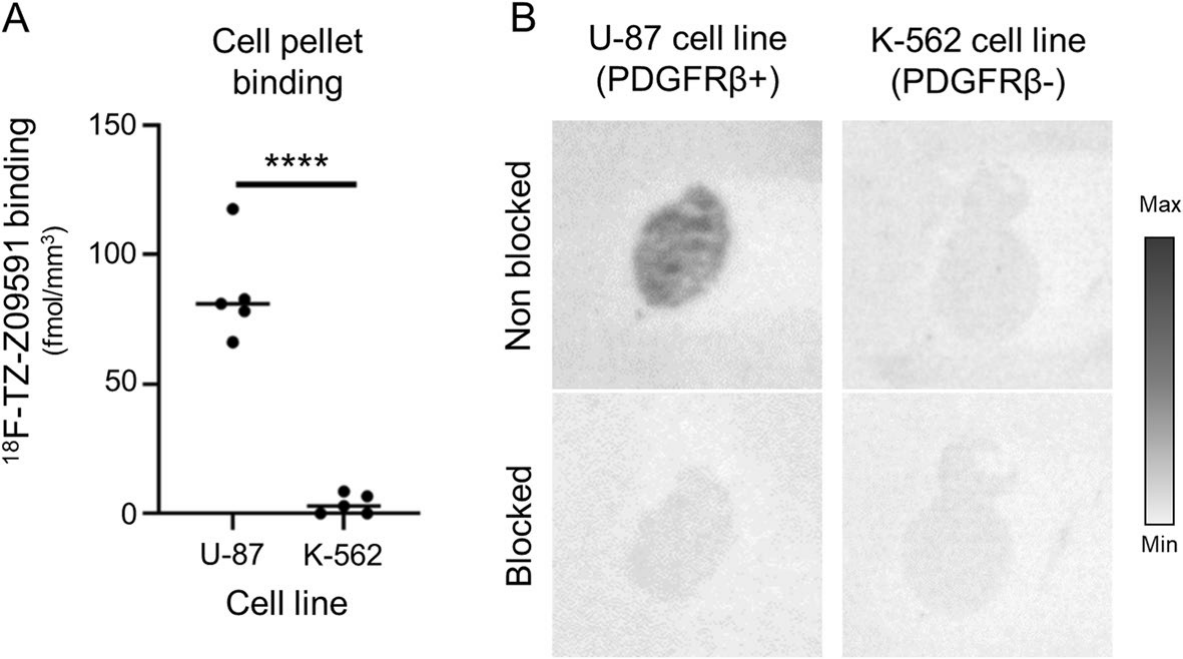
[^18^F]TZ-Z09591 uptake in cell lines. **A** Tracer uptake in U-87 cells (expressing PDGFRβ) (*n* = *5*), and K-562 cells (no PDGFRβ expression) (*n* = *5*). **B** Representative autoradiography of [^18^F]TZ-Z09591 images of U-87 cells, K-562 cells incubated with [^18^F]TZ-Z09591 alone (non blocked) or pre-treated with Z09591 (blocked). **** p < 0.0001

In vitro ARG of K-562 and U-87 cells showed that [^18^F]TZ-Z09591 uptake was detectable in U-87 cells expressing the target PDGFRβ, while no uptake was visible in K-562 cells not expressing it (Fig. 2A,B). Moreover, when the U-87 cell line was pre-treated with Cys-Z09591 in excess, then [^18^F]TZ-Z09591 uptake was blocked and binding was significantly reduced (Fig. 2B).

**Fig. 3:**
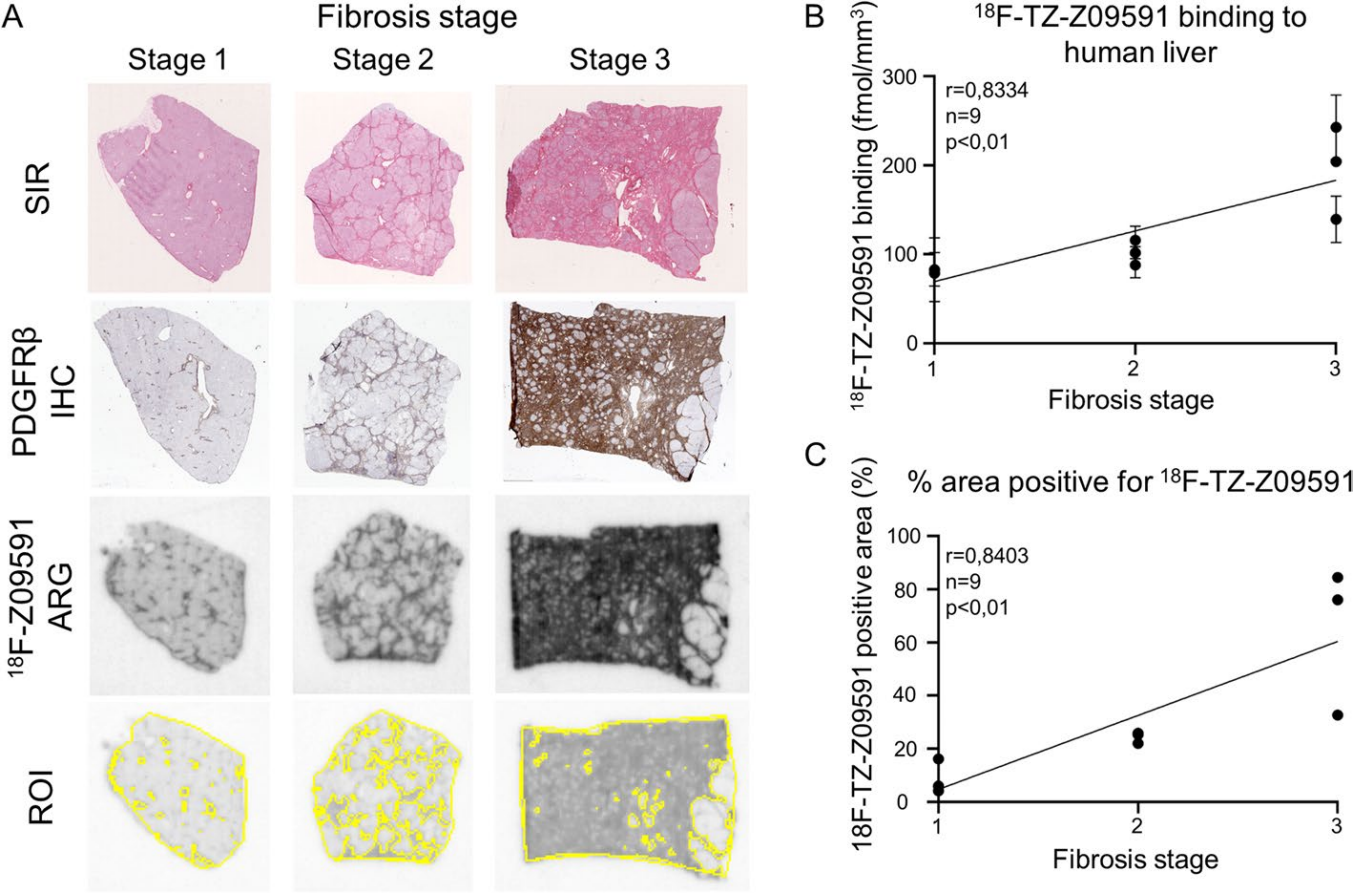
Specificity of [^18^F]TZ-Z09591 in human liver biopsies. **A** Representative Sirius red staining, PDGFRβ immunostaining, autoradiography of [^18^F]TZ-Z09591, and defined region-of-interest (ROIs) images of human liver biopsies of different fibrosis stages. **B** Correlation of [^18^F]TZ-Z09591 uptake in human liver cryosections measured by autoradiography with a fibrosis score estimated by Sirius Red staining (r = 0,8334; p < 0.01). **C** Correlation of [^18^F]TZ-Z09591 positive area in human liver cryosections measured by autoradiography with the fibrosis score estimated by Sirius Red staining (r = 0,8403; p < 0.01). *n* = *9*. SIR, Sirius red; IHC, immunohistochemistry; ARG, autoradiography; ROI, region-of-interest

The ARG of fibrotic human liver cryosections demonstrated a distinct heterogeneous uptake of radioactivity. The uptake of [^18^F]TZ-Z09591 in the ARG had a similar pattern to collagen deposition seen with SIR staining and PDGFRβ immunostaining in the different fibrosis stages (Fig. 3A). A high correlation between [^18^F]TZ-Z09591 binding in human liver biopsies or [^18^F]TZ-Z09591 positive area and histological fibrosis stages (r = 0,8334; p < 0,01, or r = 0,8403; p < 0,01) was observed (Fig. 3B, C).

### Pharmacokinetics in healthy rats

**Fig. 4:**
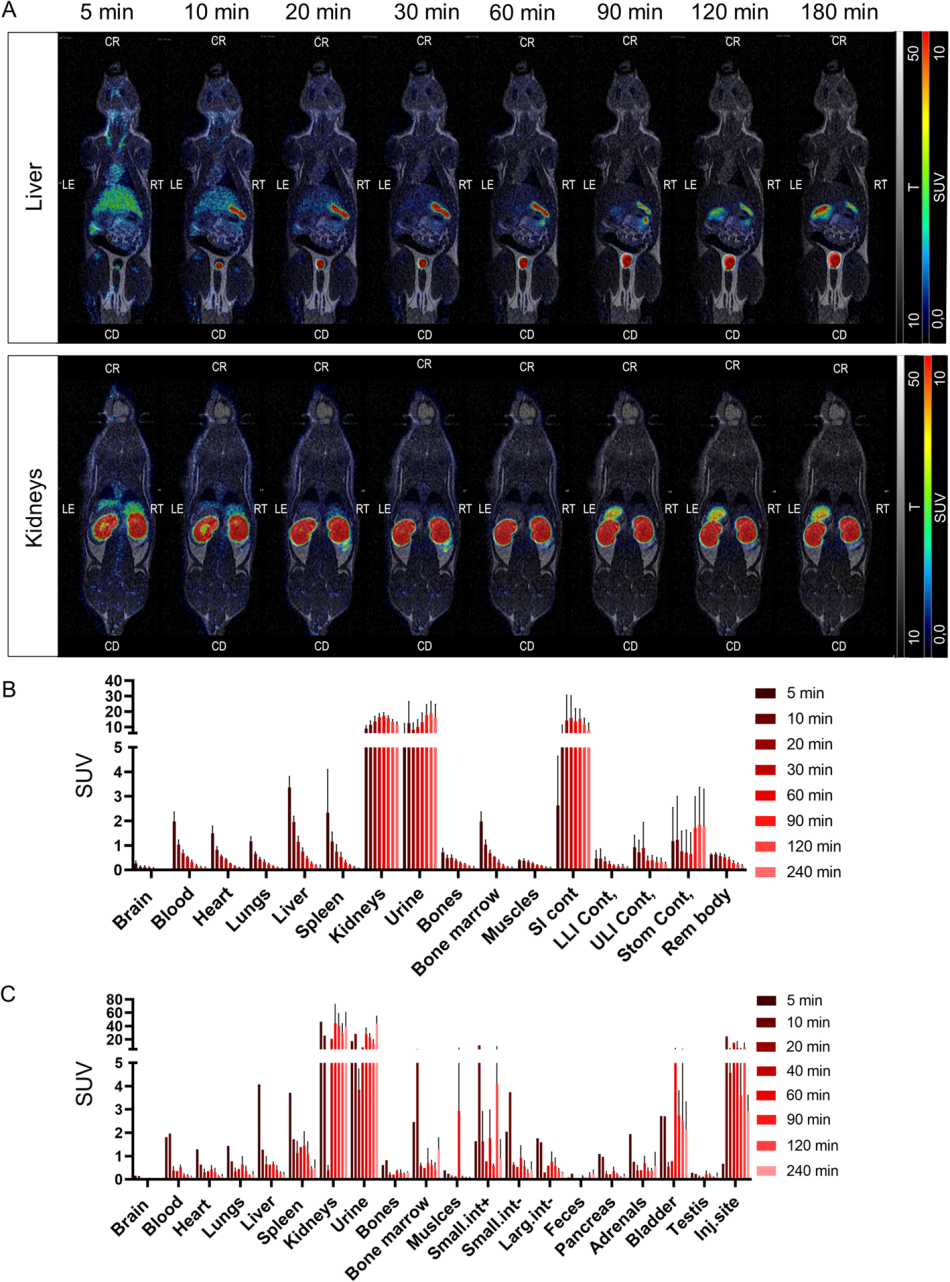
PET/MRI images of rats, in vivo*,* and ex vivo biodistribution. **A** Representative nanoPET/MRI images from 5 to 180 min post-injection in a healthy Sprague–Dawley rat, coronal sections. **B** [^18^F]TZ-Z09591 in vivo biodistribution based on PET images (*n* = *4*). **C** Biodistribution by ex vivo gamma-well counting of organs from healthy Sprague–Dawley rats (*n* = *1–2* per time point). Data are expressed as SUV or SUV ± 1 SD when n > 1. SUV, standardized uptake value

The ex vivo biodistribution (Fig. 4C) in healthy rats showed that [^18^F]TZ-Z09591 exhibited fast blood clearance with SUV values below one after 20 min. Kidney and urine uptakes were elevated at all time points. This was also observed in PET images (Fig. 4A, B). Liver uptake was high at an early time with a SUV of 4,07 at 5 min (Fig. 4C). However, the liver uptake decreased with time, at 20min the observed SUV was 0,64 ± 0,34, and 0,20 ± 0,10 at 240 min. This observation was the same as that obtained using dynamic PET (*n* = *4*) (Fig. 4A, B). In vivo biodistribution based on dynamic PET shows that the liver revealed a decreasing uptake, from an SUV of 3,37 ± 0,39 at 5 min to 0,14 ± 0,05 at 152 min.

The predicted human dosimetry was extrapolated from the ex vivo biodistribution data. The effective dose of [^18^F]TZ-Z09591 for human males was calculated to 0.015 mSv/MBq. The individual tissue with the highest absorbed dose was kidney (0.089 mGy/MBq). Assuming a limit of effective dose of 10 mSv per year in research studies in adults above the age of 18 years (Swedish regulations), it would potentially be acceptable to administer > 1300 MBq [^18^F]TZ-Z09591 per year (sufficient for repeated scanning).

### Evaluation in a fibrotic liver model

After six weeks of CCl_4_ administration, fibrosis, and infiltration of inflammatory cells in the portal areas were observed. SIR staining was performed on liver sections showing collagen deposition in the portal area. IHC targeting PDGFRβ appeared to be located in the same portal region as collagen deposition. Moreover, the collagen-rich areas in CCl_4_ mouse livers seemed to contain many macrophages, as revealed by CD68 staining. NES immunostaining was more pronounced in fibrotic livers than in healthy livers (Fig. S4).

**Fig. 5:**
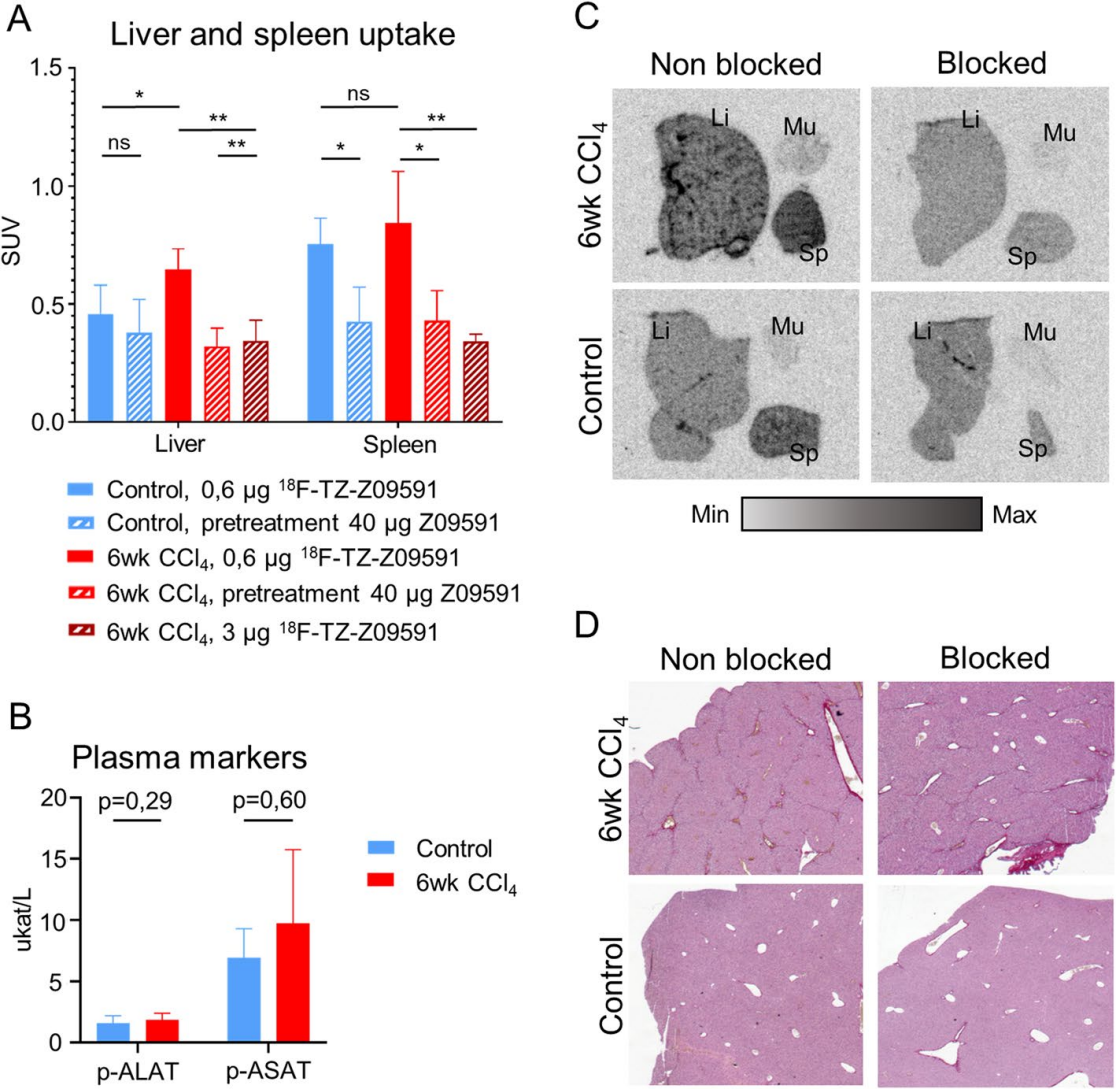
[^18^F]TZ-Z09591 uptake in 6wk CCl_4_ mice. **A** Liver and spleen uptake of [^18^F]TZ-Z09591 (at a concentration of 0,6 µg or 3 µg) and blocking study, in 6 weeks CCl_4_ treated mice compared to control mice. **B** Alanine aminotransferase (p-ALAT) and aspartate aminotransferase (p-ASAT) levels in the plasma of CCl_4_ mice compared to control mice. **C** Ex vivo autoradiograms of [^18^F]TZ-Z09591 binding, and blocking study, in the liver (Li), muscle (Mu), and spleen (Sp). **D** Representative Sirius Red staining images of liver from CCl_4_ treated and control mice (5.0 × magnification). Results from six weeks CCl_4_ treated mice are represented in red (*n*_*CCl4*_ = *13*, including *n* = *4* 0,6 µg [^18^F]TZ-Z0959, *n* = *4*, 3 µg [^18^F]TZ-Z0959, and *n* = *5* pretreated mice) and from control mice in blue (*n*_*ctrl*_ = *9*, including *n* = *4* 0,6 µg [^18^F]TZ-Z0959 and *n* = *2* pretreated mice). Data are expressed as mean ± 1 SD. * means p < 0.05 and ** means p < 0.01. CCl_4_, carbon tetrachloride; SUV, standardized uptake value; p-ALAT, plasma alanine aminotransferase; p-ASAT, plasma aspartate transaminase

Ex vivo biodistribution (Fig. 5A) showed an uptake in the fibrotic liver of SUV 0,62 ± 0,08 when 0,6 µg of [^18^F]TZ-Z09591 was injected. This uptake was significantly higher than that in healthy livers (p = 0,02). Moreover, [^18^F]TZ-Z09591 uptake in the fibrotic liver could also be blocked. Indeed, when mice were pretreated with 40 µg of Affibody molecule Z09591 alone, the uptake decreased significantly (p = 0,002). A blocking effect was also observed when [^18^F]TZ-Z09591 was injected at a higher concentration (p = 0,006). However, no difference was observed between the liver uptake of healthy mice when the Affibody molecule alone was injected in excess. In the spleen, no difference was observed between healthy and CCl_4_ mice when the radiotracer was injected at a concentration of 0,6 µg. Both uptake levels were blocked when the mice received pretreatment with Z09591. PDGFRβ staining revealed that the target was expressed in the spleens of both healthy and CCl_4_ mice (Fig. S7).

The concentrations of ALT and AST in plasma between CCl_4_ mice and control mice were not shown to be different (Fig. 5B).

Finally, ex vivo ARG consistently demonstrated heterogeneous patterns of [^18^F]TZ-Z09591 binding in the fibrotic liver corresponding to fibrotic scars (Fig. S6). Similar to the ex vivo biodistribution, when the mice received 40µg of Affibody molecule Z09591, the uptake was blocked and the heterogeneous pattern was no longer observable in the fibrotic liver. This blocking effect was also observed in in vitro ARG (Fig. S6). While a high uptake was seen in the spleens of both CCl_4_ and control mice. This uptake was also blocked in the pretreated mice. In the negative control organ, the muscle, no uptake was observed in any of the different conditions (Fig. 5C,D). Results from in vitro ARG on livers from three weeks CCl_4_ mice and scored for fibrosis stage, showed that [^18^F]TZ-Z09591 binding correlates with fibrosis score (Fig. S5).

## Discussion

Currently, liver fibrosis evaluation relies on invasive methods that are not adapted for longitudinal monitoring or non-invasive methods that are not sensitive or specific enough to detect early fibrosis. Existing liver fibrosis may be detected non-invasively by e.g. Transient Elastography (FibroScan) or Magnetic Resonance Elastography (MRE), even though their sensitivity is sub-optimal at early fibrosis stages. On the other hand, there exist no validated non-invasive methods for the identification of ongoing fibrogenesis, but such a technique could significantly help in the development of new therapies as well as early diagnosis(Friedman and Pinzani 2022; Liedtke et al. 2013).

Importantly, the development and resolution of liver fibrosis is a complex interplay between both fibrogenesis (deposition of ECM), existing levels of fibrosis (historical collagen depositions), and fibrolysis (clearance of ECM). Different interventions may affect only one of fibrogenesis and fibrolysis, and thus may not impact the same biomarkers. Here, we propose that a sensitive molecular imaging tool to quantify the level of activation of HSCs could provide a unique technology to non-invasively detect ongoing fibrogenesis.

Previous studies have demonstrated that PDGFRβ is a biomarker for fibrogenesis(Marra et al. 1994; Breitkopf et al. 2005). A recent study showed the diagnostic value of soluble PDGFRβ levels in liver fibrosis independently of the etiology of liver disease(Lambrecht et al. 2019). Hence, in this study, we investigate the potential of the radiolabeled Affibody molecule [^18^F]TZ-Z09591 targeting PDGFRβ to detect fibrogenesis in liver fibrosis.

It was previously reported that the Affibody molecule Z09591 has a dissociation constant (K_D)_ of 0.4–0.5 nM for human PDGFRβ and 6–7 nM for murine PDGFRβ(Lindborg et al. 2011). This value was obtained using a larger version of the Affibody molecule including restriction sites and a Histidine tag. In our experiment, we analyzed the minimized Affibody molecule conjugated with a TCO group (TCO-Z09591), and similar K_D_ were observed (K_D_ = 0,21 nM and K_D_ = 1,39 nM towards human and murine PDGFRβ respectively) (Fig. S3A,B and Table S3, S4). In addition, this molecule showed no affinity for platelet-derived growth factor receptor alpha (PDGFRα) (Fig. S3C). Thus, the minimized Z09591 retained or even increased the strength of interaction towards PDGFRβ. Furthermore, the addition of a TCO did not have an impact on the sensitivity of the molecule. Z09591 conjugated with TCO was then successfully labeled with [^18^F]MeTz by click chemistry and in vitro stability tests performed in human and rat plasma demonstrated the high stability of the resulting radiotracer [^18^F]TZ-Z09591, with more than 85% intact radiotracer after 3 h (Additional file 1: Fig. S2).

We evaluated this radiotracer in vitro on cells expressing PDGRFβ, in tissue from human fibrotic livers, and in vivo in a murine liver fibrosis model. [^18^F]TZ-Z09591 uptake was visible only in cells expressing the target PDGRFβ, and this uptake could be blocked by pretreatment with cold Z09591 in excess, demonstrating the specificity of the radiotracer for its target (Fig. 2).

To evaluate the translational potential of [^18^F]TZ-Z09591, we performed in vitro ARG on human fibrotic livers. The results of this experiment showed that the [^18^F]TZ-Z09591 uptake corresponded to SIR staining and PDGFRβ immunostaining (Fig. 3A). It also correlated with the fibrosis score as evaluated by a pathologist (Fig. 3B, C). Therefore, we can conclude that the radiotracer specifically binds to PDGFRβ localized in the vicinity of fibrotic scars in clinical fibrotic liver samples.

The biodistribution of [^18^F]TZ-Z09591, assessed by ex vivo organ distribution and PET imaging in healthy control rats, was a primordial step in the evaluation of the radiotracer as it allows to study its pharmacokinetics in a healthy organism. This is the first step in the in vivo evaluation and it is crucial to discover the excretion route of the radiotracer evaluated and to determine if the background uptake in an organ of interest is favorable for the use of the radiotracer to assess fibrogenesis in fibrotic tissue. The results obtained on healthy rats injected with [^18^F]TZ-Z09591 demonstrated that it was favorable for imaging fibrogenesis happening within the liver or in other organs, such as the lungs, heart or brain. Except for the kidney and urine, all analyzed organs showed rapid clearance and low background binding (Fig. 4). The high uptake in the kidneys and urine reflect a fast and main excretion route of radioactivity through the kidneys to urine. This is expected for Affibody molecules. This high kidney uptake indicates that [^18^F]TZ-Z09591 cannot be used to image kidney fibrosis in its current formulation (Fig. 4A). Dosimetry calculation demonstrated that [^18^F]TZ-Z09591 can be used for repeated scanning (e.g. enabling follow up of intervention) also in sensitive populations such as young adults. In older populations, it is likely that [^18^F]TZ-Z09591 can be administered in even higher doses, enabling more than two scans also in research studies.

We evaluated [^18^F]TZ-Z09591 in a preclinical liver fibrosis model. Ex vivo biodistribution performed on the 6wk CCl_4_ mice showed high and specific uptake in the liver. Interestingly, when 3µg of the [^18^F]TZ-Z09591 was injected into mice, a blocking effect was observed. This shows that the blocking was rapid, as well as demonstrating that the tracer is a very potent molecule. It also indicates that the peptide mass dose will be a very important parameter to consider in future preclinical and clinical studies. Liver uptake specifically corresponds to PDGFRβ immunopositive areas in or close to fibrotic scars where collagen is seen as the ARGs show (Fig. 5C,D; Fig. S5B,C and Fig. S6). Similar to that in human tissue, the uptake correlated with the fibrotic score (Fig. S5A). Specific uptake was also observed in the spleen of both CCl_4_ and healthy mice (Fig. 5A). PDGFRβ is indeed expressed physiologically in the mouse spleen, as shown by PDGFRβ immunostaining in spleens of healthy and CCl_4_ mice (Fig. S7). Moreover, [^18^F]TZ-Z09591 could detect fibroblast activation before the plasma markers AST and ALT (Fig. 5B) were conclusively positive.

Although the CCl_4_ model is a well-established model for studying liver fibrosis, the pathogenesis of this model is not similar to that of human steatotic liver disease (SLD), which rather is driven by metabolic dysfunction. However, the CCl_4_ model is still an acceptable model for liver fibrogenesis and fibrosis as the collagen deposition mechanism is similar to humans. Furthermore, it is a relatively rapid model (fibrosis arising in weeks rather than months for metabolically driven murine SLD models) (Oligschlaeger and Shiri-Sverdlov 2020). Moreover, the use of a murine model may actually underestimate the sensitivity of [^18^F]TZ-Z09591, compared to that expected in the human setting: as determined by surface plasmon resonance (SPR) the tracer affinity for human PDGFRβ is around ten times higher than that for murine PDGFRβ. This was also suggested by our results on human fibrotic liver tissue. Therefore, Z09591-based radiotracers has good potential for translation into clinical studies.

The biodistribution of [^18^F]TZ-Z09591 in healthy rats assessed by both ex vivo gamma counter and PET/MRI imaging revealed that the radiotracer potentially could be used to image fibrosis not only in the liver but also in many other organs, given the low background seen in healthy rodents, and the important role of PDGFRβ in other fibrotic diseases (Klinkhammer et al. 2018; Hudkins et al. 2004; Zymek et al. 2006; Buhl et al. 2016; Aono et al. 2014; Klareskog et al. 1990; Fellström et al. 1989). We also demonstrated that the radiotracer is sensitive for the detection of fibrosis progression in both murine and human liver tissues, as seen in the CCl_4_ mouse model and autoradiography of human fibrotic liver sections, respectively. Therefore, [^18^F]TZ-Z09591 is a promising candidate radiotracer for PET imaging of PDGFRβ also in humans.

Chronic liver diseases (CLDs) are a global burden and are estimated to impact 1,5 billion persons worldwide. CLDs and cirrhosis complications are substantial and their prevalence is rising.(Moon et al. 2020; Cheemerla and Balakrishnan 2021) The number of patients suffering from cirrhosis might be underestimated as apparently healthy subjects can be found to have advanced fibrosis and cirrhosis (Trifan et al. 2023). Many more could have ongoing fibrogenesis with early fibrosis without any way to detect it yet since there are no available methods to assess fibrogenesis. If implemented in clinical use, PET scan using radiotracer like [^18^F]TZ-Z09591 could provide a novel non-invasive technique for direct visualization of aHSCs and ongoing fibrogenesis, which could benefit the 1,5 billion persons suffering from CLD and all the other without any symptoms of their ongoing fibrogenesis. This may be beneficial for the development of novel anti-fibrotic medicines, as well as a valuable predictive diagnostic tool for improving the management of patients suffering from liver fibrosis. However, the current high price of PET scans could impede more widespread adoption, and it cannot be applied for the moment as a screening test for a large population. But, if the price for a liver biopsy is estimated to be between 1000 to 2000$ (Sanai and Keeffe 2010), a PET scan using [^18^F]TZ-Z09591 tracer would probably cost two to three times more. Nevertheless, liver biopsies carry extra cost if complications appear, on average this cost is 4579$ but its range is from 1164 to 29,641$ (Myers et al. 2008). Whereas PET scans as a non-invasive technique carry no risk of severe complications. There is a high demand for non-invasive methods and precision diagnostics, especially with the rise in the prevalence of chronic illnesses throughout the world. Therefore, PET scan availability is rising (Eurostat. 2022; Hricak et al. 2021; International Atomic Energy Agency. IMAGINE - PET scanners), thanks in particular to the increase in the number of cyclotrons in operation which can produce radionuclides such as ^18^F (International Atomic Energy Agency. Cyclotrons used for Radionuclide Production [Internet] 2023), but also the technological advances in PET systems and artificial intelligence (Alexander et al. 2019; Aide et al. 2021; Katsari et al. 2021) allowing for greater accuracy and confidence in clinical decision-making. These recent trends suggest that PET scans will play an increasing role in medicine going forward.

## Conclusion

In summary, our data describe the evaluation of the PDGFRβ binding Affibody molecule Z09591 in the context of liver fibrosis. This strongly suggests that the PET tracer [^18^F]TZ-Z09591 specifically visualizes PDGFRβ expression, and thus aHSCs responsible for fibrogenesis, in a preclinical liver fibrosis model as well as in human tissue. Therefore, [^18^F]TZ-Z09591 appears to be a suitable candidate for PET imaging of liver fibrogenesis. Further prospective studies are required to evaluate the clinical utility of PET imaging of PDGFRβ expression using the [^18^F]TZ-Z09591 for the clinical assessment of liver fibrosis progression or regression based on these promising preliminary results.

### Supplementary Information

**Additional file 1.** Contains supplementary materials, methods and results.

## Data Availability

The datasets generated during and/or analyzed during the current study are available from the corresponding author on reasonable request.

## Abbreviations

aHSC: Activated HSC
ALT: Alanine aminotransferase
APRI: Aspartate aminotransferase/platelet ratio index
ARG: Autoradiography
ARRIVE: Animal Research Reporting of In Vivo Experiments
AST: Aspartate aminotransferase
BSA: Bovine serum albumin
CCl_4_: Carbon tetrachloride
CD68: Cluster of differentiation 68
CLD: Chronic liver disease
DOTA: Dodecane tetraacetic acid
ECM: Extracellular matrix
ELISA: Enzyme-linked immunoassay
FIB-4: Fibrosis Index Based on 4 Factors
H&E: Hematoxylin and eosin
HPLC: High performance liquid chromatography
HRP: Horseradish peroxidase
HSC: Hepatic stellate cell
IHC: Immuno-histochemistry
K_D_: Dissociation constant
MRE: Magnetic Resonance Elastography
MRI: Magnetic resonance imaging
NES: Neutrophil elastase
OCT: Optimal cutting temperature compound
PFA: Paraformaldehyde
PBS: Phosphate Buffered Saline
PDGFRα: Platelet-derived growth factor receptor alpha
PDGFRβ: Platelet-derived growth factor receptor beta
PEG_3_: Tris-polyethylene glycol
PET: Positron emission tomography
ROI: Region-of-interest
RPMI: Roswell Park Memorial Institute
SD: Standard deviation
SIR: Sirius red
SLD: Steatotic liver disease
SPR: Surface plasmon resonance
SUV: Standardized uptake
TCO: Trans-cyclooctene
TLC: Thin-layer chromatography
TZ: Tetrazine
