# Supplementary material for: Imaging of fibrogenesis in the liver by [18F]TZ-Z09591, an Affibody molecule targeting platelet derived growth factor receptor β
Source: EJNMMI Radiopharm Chem. 2023 Sep 21;8:23. doi: 10.1186/s41181-023-00210-6 (PMC10513984; doi:10.1186/s41181-023-00210-6)
Supplement: Supplementary file 1 — Additional file 1. Contains supplementary materials, methods and results. [file 41181_2023_210_MOESM1_ESM.docx]

**Imaging of fibrogenesis in the liver by [^18^F]TZ-Z09591, an Affibody molecule targeting Platelet Derived Growth Factor Receptor β**

**Authors:** Olivia Wegrzyniak^1^, Bo Zhang^1^, Johanna Rokka^2^, Maria Rosestedt^1^, Bogdan Mitran^1,3^, Pierre Cheung^1^, Emmi Puuvuori^1^, Sofie Ingvast^2^, Jonas Persson^1,4^, Helena Nordström^6^, John Löfblom^4^, Fredrik Pontén^2^, Fredrik Frejd^2,5^, Olle Korsgren^2^, Jonas Eriksson^1*^, Olof Eriksson^1,3*^

**Table of contents**

Supplementary methods…………………………………………………………………..……2

Fig.S1…………………………….…………………………………………………………….3

Fig.S2…………………………………………………………………………………………..4

Fig.S3…………………………………………………………………………………………..5

Fig.S4…………………………………………………………………………………………..5

Fig.S5…………………………………………………………………………………………..6

Fig.S6…………………………………………………………………………………………..6

Fig.S7…………………………………………………………………………………………..7

Table S1………………………………………………………………………..………………7

Table S2………………………………………………………………………..………………7

Table S3…………………………………………………………..……………………………8

Table S4………………………………..………………………………………………………8

**Supplementary methods**

***In vitro plasma stability assay***

An *in vitro* stability assay was performed by mixing [^18^F]TZ-Z09591 (50 µL) with either 150 µL of phosphate-buffered saline buffer (PBS), rat plasma, or human plasma. Plasma samples were incubated at 38°C, and PBS samples at room temperature in triplicates. After incubation, the samples were analyzed by thin-layer chromatography (TLC) (Silica RP-18, eluent 8,1mM ammonium carbonate/acetonitrile 50:50).

**Surface plasmon resonance studies.** The immobilization was made by standard procedures described by the vendor Cytiva Ab using a T200 SPR instrument. All three target proteins, rhPDGF Rβ, rhPDGF Rα, and rmPDGF Rβ were diluted to 10 µg/ml in acidic immobilization buffer and immobilized with amine coupling on a CM5-sensorchip. For the human receptors immobilization buffer was 10 mM Acetate pH 5.0 and for the murine receptor, it was 10 mM Acetate pH 4.5. In order to reduce mass-transport limitations under affinity experiments the immobilization density was kept to a low level of 400-600 RU. The instrumental buffer was HBS-EP+ (10 mM HEPES, 0.15 mM NaCl, 3 mM EDTA and 0.05% v/v Surfactant P20, pH 7.4). This buffer was maintained under both immobilization and affinity experiments. When studying the affinity of TCO-Z09591 binding to the proteins a method of multi-cycle kinetics with a broad concentration range of 3000 nM diluted in a series of 3 times to 0.15 nM, was used. This was well sufficient to reach a saturation level. In the multi-cycle kinetics method, each sample was injected over the receptor surface for 60 s and left to dissociate for 180 s with a flow rate of 30 µl/min. After injection the interaction between the sample and target was broken with a 30 s long injection of 10 mM NaOH and 1 M NaCl. In order to let the surface stabilize the surface left in buffer flow for 480 s before the start of a new sample cycle.

***In vitro* autoradiography**

The fresh frozen samples of a thickness of 20µm, and mounted on Menzel Super Frost plus glass slides (VWR) were dried, then incubated in 150mL of a solution of PBS and 1% bovine serum albumin (BSA) for 15min. Optionally, to saturate the target, 2 µM of unlabeled Cys-Z09591 was added to this solution to investigate blocking of the binding. After 15 minutes of incubation in assay buffer, with or without blocking compound, 5 nM of [^18^F]TZ-Z09591 was added (corresponding to 0.1 MBq/mL) and a one hour incubation at room temperature follows. After incubation, the sections were washed twice for one minute in cold PBS / 1% BSA solution. Finally, they were rinsed by dipping them once in cold Milli-Q water. The sections were then dried for 10 minutes at 37 °C. The sections and reference were then placed in contact with a phosphor-imaging plate overnight. Once exposure was complete, the plate was scanned by a phosphor-imager system (Amersham Typhoon IP, GE Healthcare) at a pixel size of 50 ×50 μm. The ImageJ software (ImageJ 1.45S, NIH, Bethesda, USA) was used to visualize and analyze the sections. Regions of interest (ROIs) of mouse and human liver sections, cell pellets, references, and background were drawn. The mean pixel intensity values of the tissue ROIs were corrected for the background uptake. These corrected mean values were then converted into moles based on known reference concentrations.


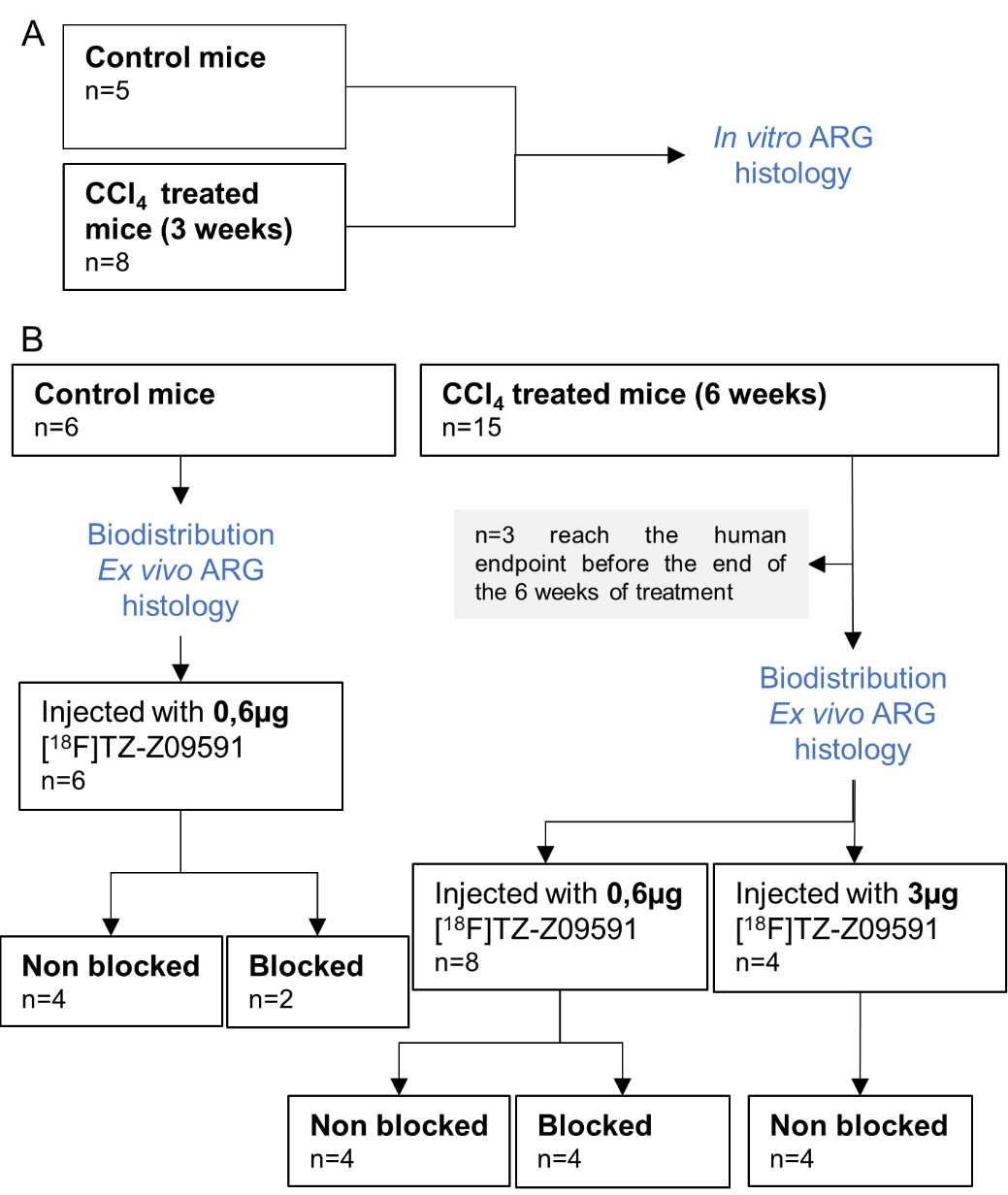


**Fig.S1. Groups from the evaluation in fibrotic liver model.** Flow chart summarizing the groups in the pilot study (A) and the full study (B).


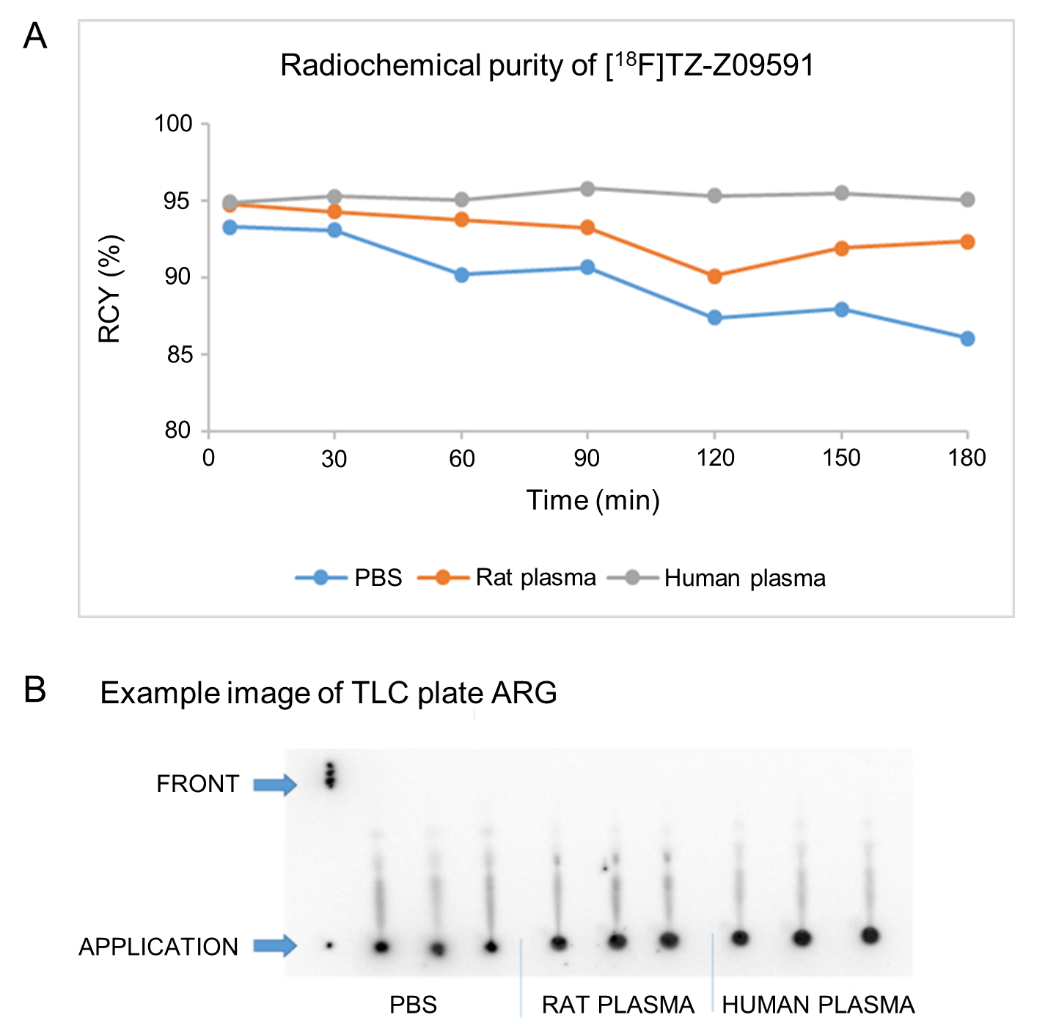


**Fig.S2. Stability of [18F]TZ-Z09591 in plasma.** Evolution of the radiochemical yields expressed in % (A), and representative radio-TLC images (B) in PBS (pH=7.4) at room temperature, rat or human serum at 38 °C over 3 h (n=3).


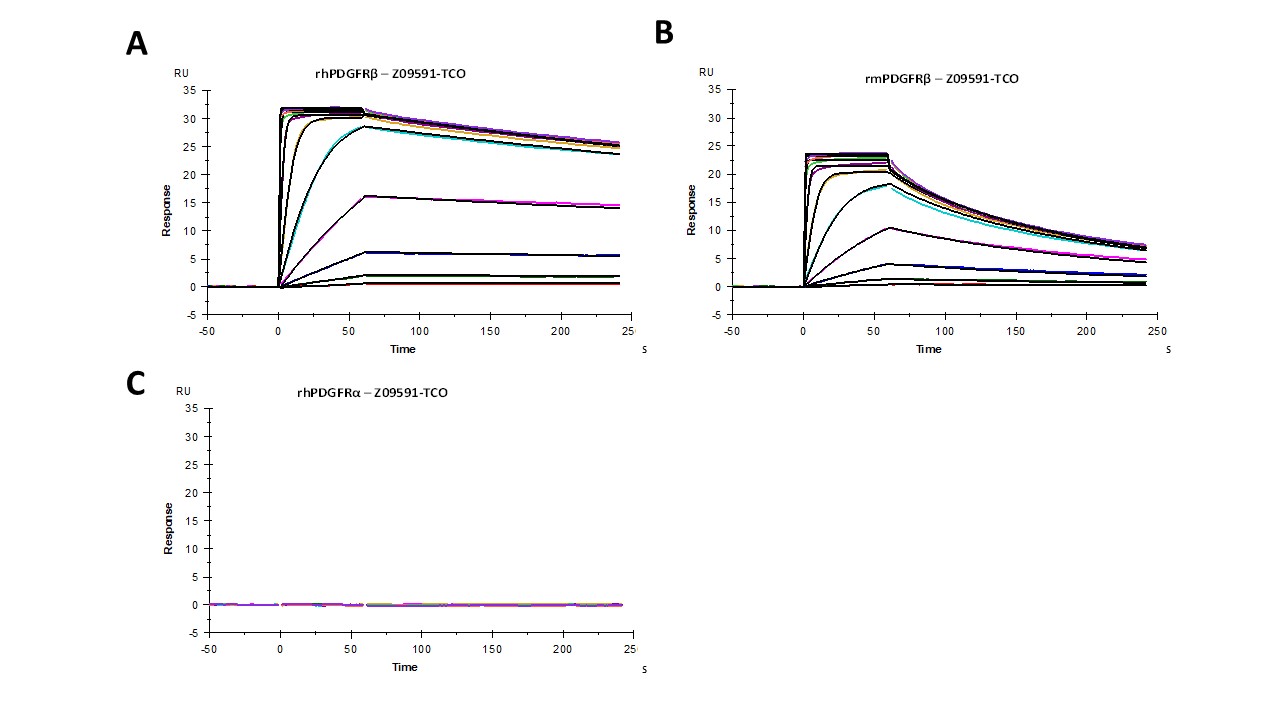


**Fig.S3. Biacore surface plasmon resonance analysis of Z09591-TCO.** Sensogram plots generated by SPR kinetic analysis demonstrate the association and dissociation characteristics between analytes (Z09591-TCO) immobilized ligand (A) human PDGRFβ (B) murine PDGRFβ and (C) human PDGRFα. RU, resonance units


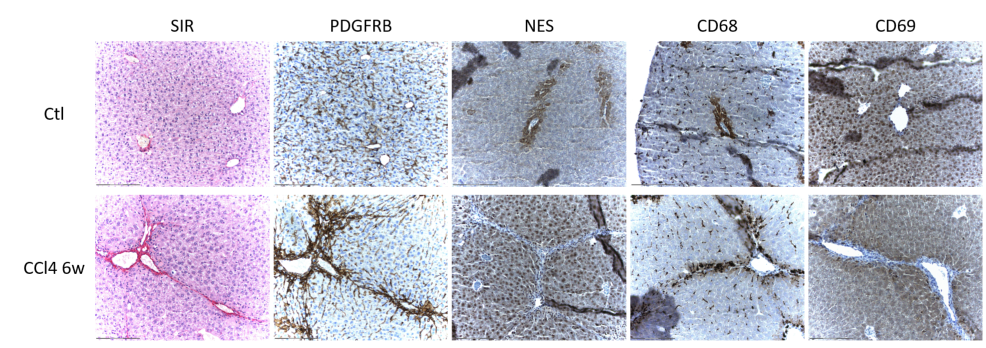


**Fig.S4. Sirius red and immunohistochemistry stainings in healthy and CCl4 mice’s liver.** Representative Sirius red staining, PDGFRB, NES, and CD68 immunostaining of paraffin liver sections from CCl4 mice (6 weeks) and healthy control are shown. (Original magnifications: 10×).


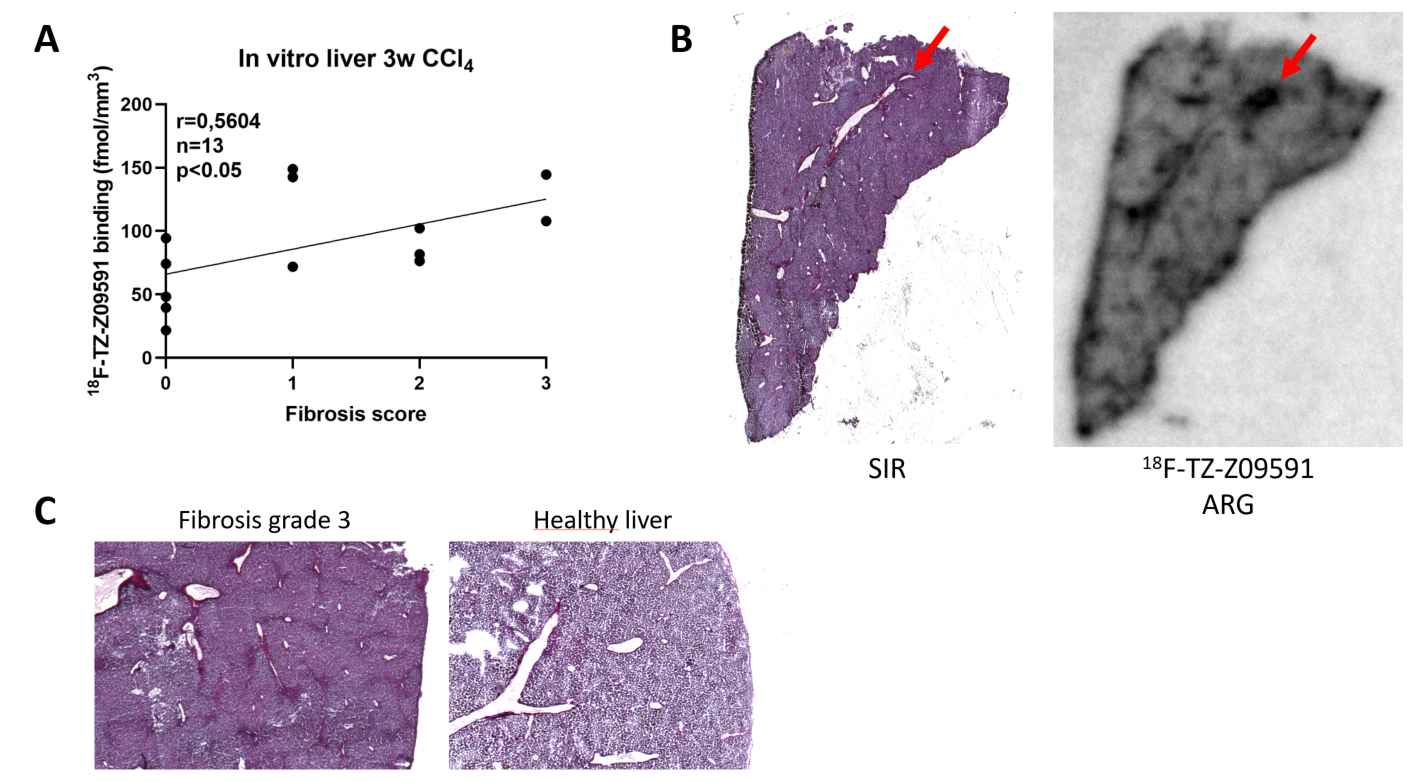


**Fig.S5. *In vitro* [^18^F]TZ-Z0959 uptake in mice treated with CCl4 for 3 weeks.** (A) Correlation of [^18^F]TZ-Z09591 uptake in mice liver cryosections measured by autoradiography with a fibrosis score estimated by Sirius Red staining (n=13; r =0,5604; p < 0.05). (B) Representative Sirius red staining, and autoradiography of [^18^F]TZ-Z0959 of a liver section from a mouse with CCl4‐induced grade 3 fibrosis. (C) Representative Sirius Red staining images (5.0x magnification) of liver sections from mice with CCl4‐induced grade 3 fibrosis and healthy controls.


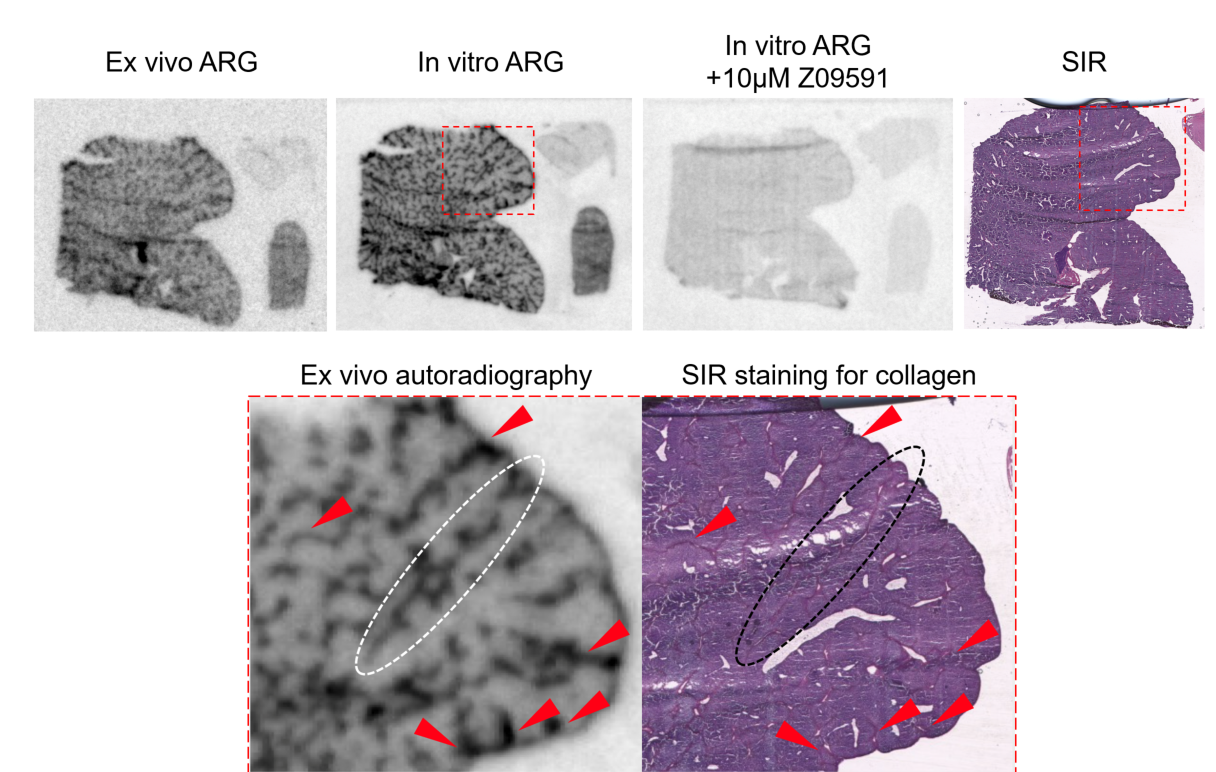


**Fig.S6. *In vitro* and *ex vivo* ARG in CCl4 mice liver.** Representative Sirius red staining, and autoradiography of [^18^F]TZ-Z0959 of a liver section from a mouse with CCl4‐induced liver fibrosis.


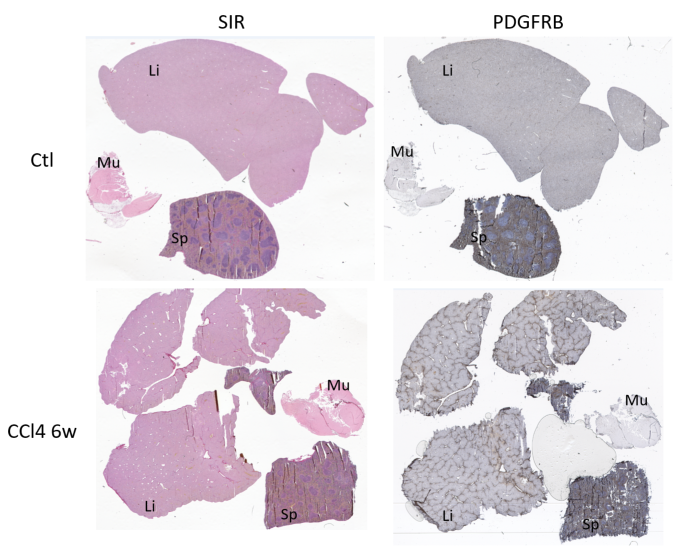


**Fig.S7.** **Sirius red and PDGFRβ stainings in healthy and CCl4 mice’s tissues.** Collagen deposition and PDGFRβ expression in healthy mice and CCl4 treated mice. Representative Sirius red staining, and PDGFRβ immunostaining of paraffin liver (Li), muscle (Mu) and spleen (Sp) sections from CCl4 mice (6 weeks) and healthy control are shown. (Original magnifications: 10×).

**Table S1. Cells used for the cell binding experiment**

| **Cells** | **Supplier** | **Catalog number** | **Organism** | **Tissue** | **Gender** | **Age** | **State** |
| --- | --- | --- | --- | --- | --- | --- | --- |
| U-87 MG | ATCC | HTB-14 | *Homo sapiens*, human | Brain | Male | / | Established |
| K-562 | ATCC | CCL-243 | *Homo sapiens*, human | Bone; Marrow | Female | 53 years | Established |

**Table S2. Antibodies used for immunohistochemistry.**

| **Antibodies against** | **description** | | **Supplier** | **Reference** | **dilution** |
| --- | --- | --- | --- | --- | --- |
| PDGFRβ | Rabbit, monoclonal [Y92] | Primary | Abcam | ab32570 | 1:300 |
| CD68 | Rabbit, polyclonal | Primary | Abcam | ab125212 | 1:100 |
| NES | Rabbit, polyclonal | Primary | Abcam | ab68672 | 1µg/ml (1:1000) |

**Table S3. SPR results for rhPDGRβ.** Results from the tri-plicate experiment showing the kinetic constants of Z09591-TCO for rhPDGRβ. ka, association constant; kd, dissociation constant; KD, equilibrium constant; Rmax, theoretical maximal response for analyte binding; RU, resonance units.

| **Experiment** | **Sample** | **ka (1/Ms)** | **kd (1/s)** | **KD (M)** | **Rmax (RU)** |
| --- | --- | --- | --- | --- | --- |
| 1 | Z09591-TCO | 5,11E+06 | 1,21E-03 | 2,36E-10 | 30,9 |
| 2 | Z09591-TCO | 7,31E+06 | 1,13E-03 | 1,55E-10 | 23,1 |
| 3 | Z09591-TCO | 4,98E+06 | 1,16E-03 | 2,33E-10 | 23,6 |
|  | Average | 5,80E+06 | 1,17E-03 | 2,08E-10 |  |
|  | SD | 1,31E+06 | 4,04E-05 | 4,59E-11 |  |

**Table S4 SPR results for rmPDGRβ.** Results from the tri-plicate experiment showing the kinetic constants of Z09591-TCO for rmPDGRβ. ka, association constant; kd, dissociation constant; KD, equilibrium constant; Rmax, theoretical maximal response for analyte binding; RU, resonance units.

| **Experiment** | **Sample** | **ka (1/Ms)** | **kd (1/s)** | **KD (M)** | **Rmax (RU)** |
| --- | --- | --- | --- | --- | --- |
| 1 | Z09591-TCO | 6,47E+06 | 9,27E-03 | 1,43E-09 | 21,1 |
| 2 | Z09591-TCO | 9,45E+06 | 1,06E-02 | 1,12E-09 | 14,3 |
| 3 | Z09591-TCO | 6,21E+06 | 9,97E-03 | 1,61E-09 | 13,1 |
|  | Average | 7,38E+06 | 9,95E-03 | 1,39E-09 |  |
|  | SD | 1,80E+06 | 6,65E-04 | 2,48E-10 |  |
